# The Effects of Low-Nickel Diet Combined with Oral Administration of Selected Probiotics on Patients with Systemic Nickel Allergy Syndrome (SNAS) and Gut Dysbiosis

**DOI:** 10.3390/nu12041040

**Published:** 2020-04-09

**Authors:** Francesca Lombardi, Fabiana Fiasca, Martina Minelli, Dominga Maio, Antonella Mattei, Ilaria Vergallo, Maria Grazia Cifone, Benedetta Cinque, Mauro Minelli

**Affiliations:** 1 Department of Life, Health & Environmental Sciences, University of L’Aquila, Via Pompeo Spennati, Building Rita Levi Montalcini, Coppito, 67100 L’Aquila, Italy; francesca.lombardi@univaq.it (F.L.); fabiana.fiasca@univaq.it (F.F.); antonella.mattei@univaq.it (A.M.); mariagrazia.cifone@univaq.it (M.G.C.); 2 PoliSmail, Specialistic Unit Allergic & Immunological Pathologies, Via Clemente Rebora, 1, 73100 Lecce, Italy; martinaminelli@polismail.it (M.M.); dmaio@polismail.it (D.M.); ivergallo@polismail.it (I.V.); 3 Pegaso Online University, Centro Direzionale Isola F2, 80132 Naples, Italy

**Keywords:** systemic nickel allergy syndrome, SNAS, gut dysbiosis, urinary dysbiosis markers, indican, skatole, low-nickel diet, probiotics

## Abstract

Background: Nickel (Ni) oral consumption may elicit systemic reactions in patients affected by systemic nickel allergy syndrome (SNAS), including gastrointestinal symptoms, which in turn are associated with gut dysbiosis. We evaluated the effects of a low-Ni diet alone or in combination with the oral consumption of appropriate probiotics on Ni-sensitivity and urinary dysbiosis markers in SNAS patients. Methods: *n* = 51 patients with SNAS and concomitant intestinal dysbiosis were enrolled in the study. According to the urinary indican/skatole levels, quantified through a colorimetric and a high-performance liquid chromatographic method, respectively, patients were assigned to a dysbiosis type/grade and followed a low-Ni diet for three months. Along with the diet, 22 patients also consumed probiotics based on the dysbiosis type. In particular, a Lactobacilli- or Bifidobacteria-containing formulation was administered to patients with fermentative or putrefactive dysbiosis, respectively, while a broad-spectrum probiotic formulation containing both Lactobacilli and Bifidobacteria was administered to patients with mixed dysbiosis. After three months, patients were invited to repeat the Ni-stimulation and the dysbiosis tests. Results: The fermentative dysbiosis group represented the largest group followed by the mixed dysbiosis group, while only two patients had putrefactive dysbiosis. Overall, at three months of treatment in general (diet alone with or without probiotics), the Ni-sensitivity and dysbiosis levels were strongly ameliorated. The association of a low-Ni diet with a specific probiotic oral supplementation was significantly more effective in decreasing dysbiosis levels or reaching eubiosis than with diet alone. Conclusion: Our results, while confirming the benefits of a low-Ni diet in SNAS patients, strongly support that appropriate adjuvant treatment with probiotics significantly helps to improve intestinal dysbiosis or restore a healthy microbiota.

## 1. Introduction

Nickel (Ni) is a chemical element widely diffused in the environment [1]. Topical Ni exposure occurs from metallic items, household products, and cosmetics, whereas systemic exposure is possible from food, water, surgical implants, and dental materials [2]. The classical presentation of an Ni allergy is allergic contact dermatitis [2,3], a T-cell-mediated inflammatory skin disease caused by repeated skin exposure to a specific antigen in a sensitized individual [4]. In sensitized subjects, the ingested Ni-containing compounds, in addition to typical systemic cutaneous lesions, may induce gastrointestinal symptoms like those characterizing inflammatory bowel disease (IBS), i.e., nausea, pyrosis, meteorism, abdominal pain, diarrhea, and constipation. This clinical picture, known as Systemic Nickel Allergy Syndrome (SNAS) [5,6] and considered to be an emergent allergic condition, is associated with immune system dysregulation with massive infiltration of pro-inflammatory CD4+ T lymphocytes in the duodenal lamina propria and epithelium [7], as well as the involvement of Th2-type cytokines (IL-5 and IL-13) [8]. Ni systemic absorption may elicit systemic reactions, including eczematous, vasculitic, mucosal, respiratory, urticarial, and gastrointestinal symptoms. Although in the literature there is no general agreement about the efficacy of a dietary treatment [9], some reports have confirmed improvement of dermatitis with a Ni-free or low-Ni diet for a period ranging from four weeks to six months [10,11,12]. The oral hypo-sensitization reduced symptoms and induced oral tolerance to Ni with no side effects or adverse events, allowing patients to gradually and safely re-introduce Ni-rich foods, thus improving their quality of life [8,10,13].

Some studies have suggested that dietary Ni exposure can alter the equilibrium of the symbiotic intestinal microbiota [14,15]. Normal gut microbiota comprises mostly several genera of Gram-positive Firmicutes and many Gram-negative Bacteroidetes, i.e., Bacteroides, Prevotella, Parabacteroides, and Alistipes [16]. In addition, several other phyla, including the Proteobacteria, Actinobacteria, Fusobacteria, Verrucomicrobia, Archaea, Eukarya, and others, are found to be part of the human gastrointestinal microbiome. Gut microbiota imbalance has been mainly associated with the decline in Firmicutes and Bacteroidetes, and an increase in detrimental microbes [17].

Wu et al. [15] showed that dietary Ni affected the amount and diversity of intestinal microbiota in the ileum and cecum of broilers, decreasing the number of some beneficial bacteria such as *Bifidobacterium* spp. (phylum Actinobacteria) and/or *Lactobacillus* (phylum Firmicutes), while increasing bacterial species harmful to the animals. Significant changes in intestinal microbiota composition were also observed in rats exposed daily to Ni, with a dose-dependent effect [14]. These findings imply that Ni has a toxicological impact on the intestinal ecosystem and, possibly, functions. The qualitative and quantitative dysregulation of the intestinal microbiota setup is indicative of a pathologic condition called dysbiosis, which may cause symptoms of varying types and severity in immunological, metabolic, psychiatric, and intestinal diseases [18,19]. Gut dysbiosis is associated with altered microbial metabolism with a higher production and reabsorption of intestinal bacterial metabolites derived from the breakdown of tryptophan, such as indican (3-indoxyl sulfate) and 3-methyl-indole (also named skatole) [20,21]. These tryptophan catabolites, typically found in traces in urine, may provide rough indications of the enteric tract most affected, and are currently used to diagnose an intestinal dysbiosis [22,23,24,25,26]. In particular, an increase of indican levels are indicative of a fermentative dysbiosis involving mostly the small intestine; on the other hand, increased urinary levels of skatole are indicative of a putrefactive dysbiosis involving mostly the colon [23,25,27,28,29,30,31,32,33]. A combined increase of both their values underlines a “mixed” dysbiosis, involving both the small intestine and colon.

In this context, probiotics have enormous potential for modifying the gut microbiota with beneficial effects, including improved digestion, boosted immunity, and lowered risk of certain diseases. The oral assumption of probiotics is also related to a reduction in the incidence and severity of a substantial series of disorders affecting not only the gastrointestinal tract [34,35,36]. Microbes with probiotic characteristics mainly belong to the *Lactobacillus* and *Bifidobacterium* genera and are regulated as dietary supplements and food [37,38,39].

The current work aimed to evaluate the effects of a low-Ni diet alone or in combination with a probiotic assumption on both Ni-sensitivity level and urinary dysbiosis markers in subjects with SNAS. The most appropriate replanting probiotic therapy was chosen based on the type of associated dysbiosis. We show evidence that the association of a low-Ni diet with a specific probiotic oral supplementation is significantly more effective in decreasing urinary levels of dysbiosis markers than a low-Ni diet alone, thus helping to improve intestinal dysbiosis or restore a healthy microbiota.

## 2. Materials and Methods

### 2.1. Patients’ Enrolment and Exclusion Criteria

We enrolled 51 patients with clinical histories singularly characterized by complex symptomatologic pictures, not necessarily overlapping, with more frequent involvement of the gastrointestinal apparatus (abdominal bloating/tension, bowel disorders, cramping, dyspepsia, and pyrosis, nausea, and/or vomiting); cutaneous system (itching, dermographism, and hives); but also with reported reactive phenomenology of systemic significance, apparently non-specific, although recurring and at times of high intensity (headaches, diffuse arthralgia, marked asthenia, edemas, stomatitis, and relapsing ulcers). Patients of both sexes between 18–60 years of age with no race restriction were included in the sample. Patients were consecutively enrolled at the Polismail Specialistic Center (Lecce, Italy). The study was approved by the Internal Review Board of the University of L’Aquila and conducted according to the requirements of Good Clinical Practice of the European Union and the current revision of the Helsinki Declaration. Written informed consent was obtained from each participant who had voluntarily agreed to participate. We protected the privacy and anonymity of the individuals involved. Patients were chosen after undergoing an articulate procedure well defined in its progressive stages, which involved a preliminary collection of anamnesis data, aimed, among other objectives, at searching for pathologies set in differential diagnosis with SNAS (Coeliac disease, inflammatory bowel disease (IBD), neurological and dermatological pathologies) and taking into consideration, when present, the exclusion criteria for enrolment which included with Small Intestinal Bacterial Overgrowth (SIBO), active autoimmune pathologies, infections (viral, bacterial, parasitological), and clinically relevant multiple sensitivities to respiratory allergens. The second diagnostic step consisted of performing both the oral Ni stimulation test (Ni-OST) and Ni patch test. The oral provocation test versus placebo was performed in order to confirm the actual role of Ni in the genesis of the disorders. The test consisted of the administration of capsules containing increasing amounts of Ni sulfate, starting with a very low dose (0.625 mg), usually not able to provoke evident reactions. At regular intervals (60 min), increasing quantities of Ni sulfate were administered, reaching the maximum stimulation threshold (established at 5 mg), that is, until the occurrence of the reactive phenomena that somehow re-simulated the initial symptoms as described in the anamnesis by the patient and scrupulously verified through the rigorous procedures of the double blind versus placebo test. The SNAS diagnosis was confirmed only in case of the occurrence of symptoms after the oral administration of Ni and through a negative placebo test. The patch test was used to detect a lesion eventually characterized by inflammatory infiltrate, erythema, and possible blistering as unquestionable evidence of cell-mediated Ni allergy after removing a cellulose chamber containing Ni sulfate kept on the patient’s back for 48–72 h. The dysbiosis test, based on urinary quantification of two metabolites deriving from the decomposition of tryptophan, skatole (3-methyl-indole), and indican, was performed as described below.

### 2.2. Dysbiosis Test

Fresh urine samples (20 mL) were collected in the morning after fasting overnight and stored at −20 °C before the assays. Urinary levels of indican (3-indoxyl sulfate) and skatole (3-methyl-indole) were assayed before and after treatment through a colorimetric and high-performance liquid chromatographic method, respectively. For skatole determination, 300 μL of acetonitrile was added to an equal volume of each urine sample, vortexed three times for 5 s, and cooled to 4 °C for 15 min. Then samples were centrifuged for 5 min at 12,000 rpm at 1 °C. Clear supernatants were collected and used for chromatography analysis on a Shimadzu Nexera system equipped with a Lab Solutions software and a fluorescence detector programmed for excitation at 280 nm and emission at 360 nm (Shimadzu Corporation, Tokyo, Japan). Chromatographic separation was obtained on a C18 column (3 μm, 150×4.6 mm; Shimadzu Corporation) operating at 40 °C. The mobile phase was delivered isocratically, using 80% methanol and 20% H_2_O at a flow rate of 1 mL/min. All the HPLC quality solvents were from Carlo Erba (Milan, Italy). For urinary indican determination, an Indican Assay Kit (Sigma Aldrich, Saint Louis, MO, USA) was used according to the procedures indicated by the manufacturer. According to previous reports [25,30,40], urinary indican and skatole were considered normal at values lower than 10 mg/L and 10 µg/L, respectively.

Based on the reference intervals reported in Table 1, the patients were assigned to a dysbiosis grade.

### 2.3. Study Design

All patients (*n* = 51) were invited to follow a low-Ni diet which, apart from the temporary exclusion of food with an Ni concentration higher than 50 μg/Kg, also involved following specific hygienic norms for food preparation and preservation (avoiding metal containers and using glass, pyrex, Teflon and unglazed ceramic tableware). The low-Ni diet was followed for three months, after which the patients went through a new clinical analysis in order to verify any benefits obtained. As previously reported [8], in the diet the following foods were avoided: almonds, apricots, asparagus, avocados, beans, cabbages, carrots, cauliflowers, cocoa, figs, flounder, hazelnuts, herring, lentils, lettuce, lobster, maize, margarine, mushrooms, mussels, oats, onions, oysters, peanuts, peas, plaice, potatoes, prunes, raisins, shellfish, spinach, tea, tomatoes, tomato puree, walnuts, and yeast. In addition, considering that the study aimed to comparatively analyze the effects of a low-Ni diet alone and combined with probiotic administration, all patients were asked not to take milk, yoghurt, and dairy products throughout the observation period to prevent these probiotic-containing foods from affecting the detected parameters. Only one patient, included in the low-Ni diet + probiotics group, explicitly asked to take the probiotic with half a glass of milk. Of the 51 enrolled patients, 22 were available to take probiotics for three months along with the prescribed low-Ni diet. Specific probiotic therapy for each of these patients was established, consistent with the results of the dysbiosis test; the Lactobacilli-containing formulation was administered to patients with fermentative dysbiosis, while the Bifidobacteria-containing formulation was administered in the putrefactive dysbiosis group evidenced by high levels of skatole. A broad-spectrum probiotic formulation containing both Lactobacilli and Bifidobacteria was selected instead for patients with mixed dysbiosis, as assessed by abnormal levels of both urinary metabolites. After three months, patients were invited to repeat the Ni-OST and the dysbiosis test. More specifically, the following adjuvant treatment with probiotics was planned as follows: a) fermentative dysbiosis: one capsule twice daily of a probiotic formulation containing Lactobacillus casei LC18, Lactobacillus acidophilus LA3, Lactobacillus reuteri LR200, Lactobacillus rhamnosus LRH11, Lactobacillus fermentum LF350, Lactobacillus plantarum LPB22, and Streptococcus thermophilus STB32, ~40 × 10^9^ CFU/capsule (Starterflor, A.V.D Reform Srl, Parma, Italy); b) putrefactive dysbiosis: one capsule twice daily of a probiotic product containing Bifidobacterium lactis BL 04, Bifidobacterium breve BB 03, Bifidobacterium bifidum BB 06, and Bifidobacterium longum BL 05, ~4.0 × 10^9^ CFU/capsule (Bifiselle, Bromatech Srl, Milan, Italy); c) mixed dysbiosis: one capsule daily of a multi-strain probiotic containing eight strains, namely Streptococcus thermophilus DSM 24731, Bifidobacterium breve (B. breve) DSM 24732, B. longum DSM 24736, B. infantis DSM 24737, Lactobacillus paracasei (L. paracasei) DSM 24733, L. acidophilus DSM 24735, L. delbrueckii subsp bulgaricus DSM 24734, and L. plantarum DSM 24730, at 112 × 10^9^ live bacteria/capsule (Vivomixx MENDES S.A., Lugano Switzerland). Patients were instructed to keep the probiotic product at 4 °C in the refrigerator at home and to take it with one glass of water, milk, or juice at room temperature. To guarantee adherence to treatment, multiple actions were planned, i.e., behavioral, educational, integrated care through the multidisciplinary health care team, self-management, and risk communication interventions. In addition, daily reminders through phone calls, texts, and video messages aimed at improving adherence to treatment.

No evidence of notable adverse events with probiotic assumption was recorded. Occasionally, just in the early days of treatment, very few patients (n. 3, 13,6%) reported gastrointestinal symptoms including tummy and gut upset with some episodes of diarrhea or constipation of a mild grade.

### 2.4. Statistical Analysis

The characteristics of the study sample were analyzed using descriptive statistics. The discrete and nominal variables were expressed in terms of frequencies and percentages, and the continuous variables were expressed as median values and interquartile ranges (IQRs). Differences in age, sex, results of the Ni-OST and patch test, as well as pre-treatment concentrations of skatole and indican were evaluated and analyzed using the Kruskal–Wallis test for the continuous variables and the χ^2^ test for the qualitative variables, as well as the non-normal data distribution (Shapiro–Wilk test). The statistical significance between pre- and post-treatment concentrations of skatole and indican, as well as Ni-OST, were evaluated through the Wilcoxon signed-rank test. To determine the effects of the low-Ni diet with or without probiotics, we used the McNemar test for pairwise comparisons of frequencies within-groups and χ^2^ test or Fisher test for post-treatment pairwise comparisons of frequencies between-groups. Based on the median age of the sample, two age classes were built (<37 years and ≥37 years) and were compared for pre- and post-treatment values of skatole and indican; between-groups differences were evaluated using the Wilcoxon rank-sum test.

Wilcoxon rank-sum test (unpaired) or Wilcoxon signed-rank test (paired) were used to compare quantitative variables. The univariate logistic regression model was used to identify the association with reaching eubiosis, expressed as odd ratios (ORs) and 95% confidence intervals (95% CIs), using the presence or absence of eubiosis post-treatment as the dependent variable; the urinary dosages and the use or not of probiotics were considered independent variables. All the tests used were bi-directional and the level of statistical significance was fixed at 5%. The statistical analysis was carried out by using the STATA (Stata Statistical Software: Release 15–Stata Corp LP, College Station, TX, USA) statistical package.

## 3. Results

### 3.1. Characteristics of Patient Samples Stratified by Dysbiosis Type

Characteristics of the patient samples stratified for dysbiosis type are reported in Table 2. High levels of urinary indican and a normal amount of skatole, a condition associated with a fermentative dysbiosis, were revealed in 33 patients, while just two patients showed abnormal values of urinary skatole and normal indican, a condition associated with a putrefactive dysbiosis. Elevated levels of both indican and skatole, indicative of mixed dysbiosis, were detected in 16 patients. The comparative data analysis revealed statistically significant differences, as expected, between levels of both skatole and indican (*p* < 0.001 and *p* = 0.048, respectively) in the different dysbiosis patients (fermentative, putrefactive, and mixed). On the other hand, as for age and sex, no significant differences were observed in both the oral or cutaneous Ni sensitivity between the groups of patients with fermentative, putrefactive, or mixed dysbiosis, thus highlighting the high homogeneity of the groups studied.

As reported in Table 3, among the 18 patients who had positive results for the skatole test, nine patients showed grade I dysbiosis (range 11–20 µg/mL), six patients showed grade II dysbiosis (range 21–40 µg/mL), and three patients showed grade III dysbiosis (>40 µg/mL). Thirty-three patients had normal levels of skatole (range 0–10 µg/mL). On the other hand, the urinary dosage of indican revealed that only two patients had normal values (range 0–10 mg/mL), 13 patients had grade I dysbiosis (range 11–20 mg/mL), 22 patients had grade II dysbiosis (range 21–40 mg/mL), and 14 patients had grade III dysbiosis (>40 mg/mL) (Table 3).

### 3.2. Comprehensive Assessment of the Effects of Treatment with Low-Ni Diet Alone or Combined with Probiotic Assumption

**Figure 1:**
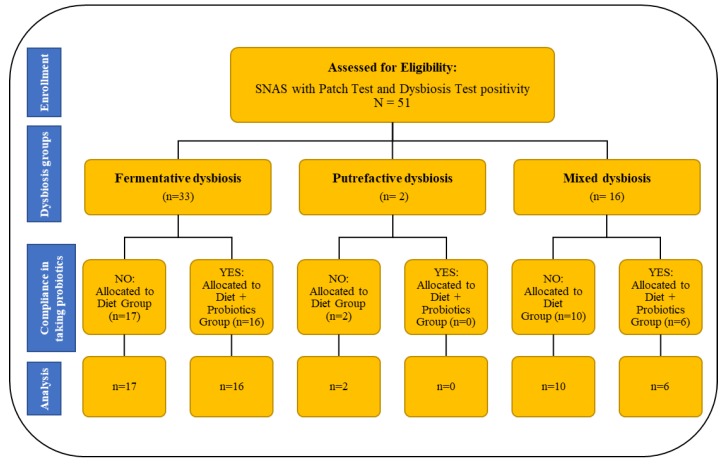
Summary of study design and patient flow diagram.

The study design and flow diagram is summarized in Figure 1, showing patients included in the study and divided into groups based on dysbiosis type and willingness to take probiotics along with the low-Ni diet. The larger group was the one with fermentative dysbiosis (66%), followed by the mixed dysbiosis group (31.4%), while only two patients had putrefactive dysbiosis. As reported in Figure 1, in the fermentative dysbiosis group 17 patients (51.5%) followed the low-Ni diet alone, while 16 patients (48.5%) agreed to go on the low-Ni diet and simultaneously take probiotics for the entire study period. In the mixed dysbiosis group, 10 patients (62.5%) followed the low-Ni diet, while six patients (37.5%) agreed to take probiotics as well. The two patients with putrefactive dysbiosis agreed to follow only the low-Ni diet.

**Figure 2:**
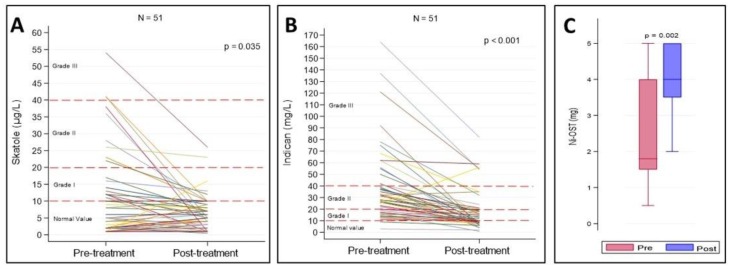
Pre- and post-treatment levels of skatole, indican, and oral Ni stimulation test (Ni-OST). Skatole and indican levels were represented by spaghetti plots (**A, B**) and the amounts of Ni used in the Ni-OST were graphed by boxplots (**C**).

At three months of treatment with the Ni-low diet alone or combined with probiotic consumption, patients were resubmitted to the dysbiosis test and to the Ni-OST. Overall, the urinary levels of skatole and indican significantly decreased in patients after treatment, as graphically shown in Figure 2A and B, respectively. The Ni sensitivity was significantly reduced after treatment as evidenced by the higher amounts of Ni needed to induce SNAS-associated symptoms when compared to pre-treatment subjects (Figure 2C). The only patient who had asked to take the probiotic with milk belonged to the mixed dysbiosis group, with a grade I dysbiosis based on the values of both indican and skatole. The evaluated parameters in this patient did not significantly differ from those of the other patients in the same group, probably due to the minimum amount of milk taken daily.

**Figure 3:**
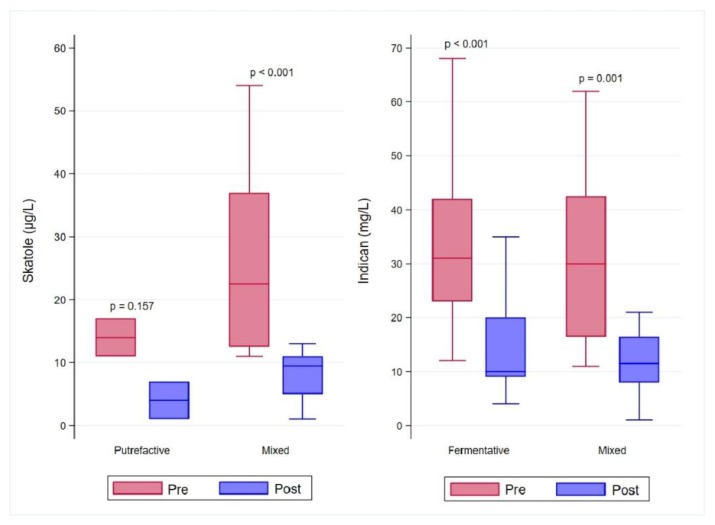
Comparison of pre- and post-treatment levels of urinary dysbiosis markers. Data are stratified for dysbiosis type based on the pre-treatment levels of skatole and indican.

As detailed in Table 4 and graphically shown in Figure 3, the urinary levels of skatole significantly decreased after treatment in patients with mixed dysbiosis. On the other hand, in patients with putrefactive dysbiosis, being only two subjects, even if the treatment led to a decrease of skatole levels, no statistical significance could be observed. The urinary concentrations of post-treatment indican turned out significantly lower either in patients with fermentative or mixed dysbiosis when compared to pre-treatment levels. The Ni sensitivity was significantly reduced after treatment as evidenced by the higher amount of Ni needed to induce symptoms when compared to pre-treated subjects (Table 4).

**Figure 4:**
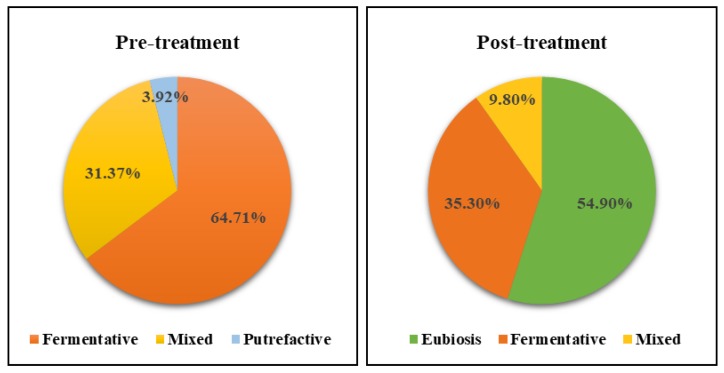
Percent distribution of dysbiosis types before and after treatment.

Overall, at three months of treatment in general (low-Ni diet alone or in combination with probiotic assumption), the percent distribution of fermentative, putrefactive, or mixed dysbiosis results were strongly ameliorated when compared to the pre-treatment profile (Figure 4). The percent of patients with dysbiosis was significantly and strongly reduced after treatment, decreasing from 100% to 54% in 90% of patients, which shifted versus eubiosis conditions. A reduction was also observed in both fermentative dysbiosis cases (from 64.71% to 35.30%) and mixed dysbiosis (from 31.37% to 9.80%).

### 3.3. Comparison between Effects of Low-Ni Diet Alone or in Combination with Probiotic Assumption

**Figure 5:**
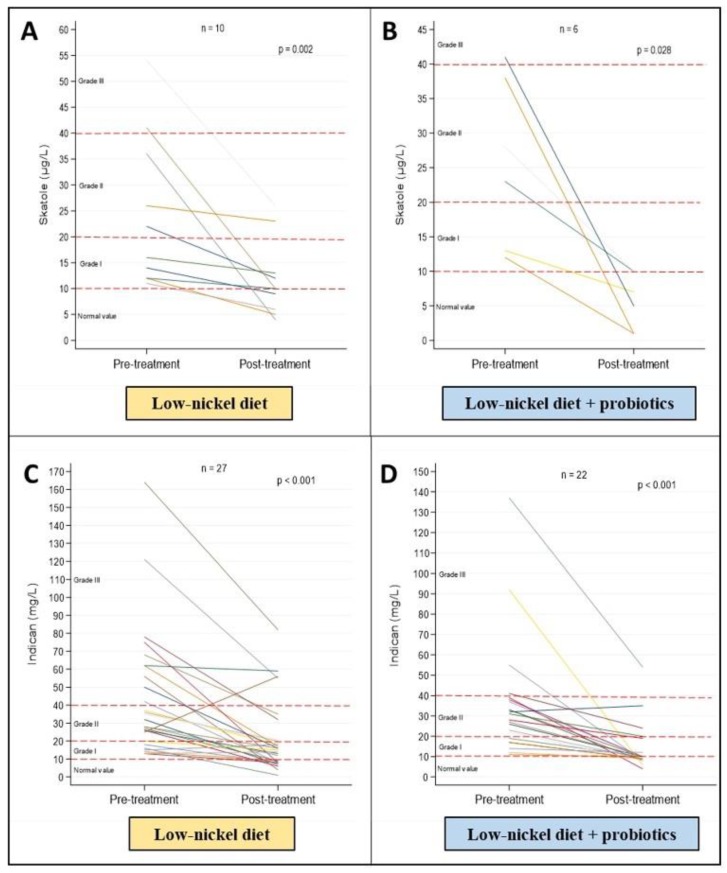
Representation of pre- and post-treatment concentrations of skatole (µg/L) and indican (mg/L) in patients with pre-treatment abnormal values by spaghetti plots. (**A**) Pre- and post-treatment levels of skatole in patients who followed the low-Ni diet alone. (**B**) Pre- and post-treatment levels of skatole in patients who followed the low-Ni diet combined with probiotics. (**C**) Pre- and post-treatment levels of indican in patients who followed the low-Ni diet alone. (**D**) Pre- and post-treatment levels of indican in patients who followed the low-Ni diet combined with probiotics.

The results of the skatole and indican dosage for each patient before and after three months of treatment with the low-Ni diet alone or combined with the probiotic consumption are shown in Figure 5. Patients with abnormal values of skatole (*n* = 10, mixed dysbiosis) who followed a low-Ni diet alone reached a significant improvement (*p* = 0.002), but in the six patients who followed the diet combined with probiotics, the improvement was greater as they shifted versus the eubiosis condition (*p* = 0.028). Concentrations of indican (mixed and fermentative dysbiosis) decreased in both groups (*p* < 0.001), but they reached lower values in patients who followed the low-Ni diet combined with probiotic consumption.

Absolute and percent distributions of patients pre- and post-treatment with only diet or diet plus probiotics, as well as comparisons of results obtained between patients shifted to eubiosis or a lower grade dysbiosis, are reported in Table 5. In detail, 12 out of 29 patients who followed only the diet and 16 out of 22 patients who followed the diet with probiotics shifted to eubiosis (*p* < 0.001, and *p* = 0.031, respectively). These differences were also observed in the comparison between the two groups (post-post treatment, *p* = 0.026). Among patients who followed the diet plus probiotics, the frequency of fermentative dysbiosis decreased to less than half (72.72% vs. 27.27%, *p* = 0.002), and mixed dysbiosis resolved in all patients (*p* = 0.031). The Ni sensitivity was significantly reduced both with the diet only and with diet plus probiotics (*p* = 0.031 and *p* = 0.028, respectively). Indeed, no post-post difference emerged between the two groups.

**Figure 6:**
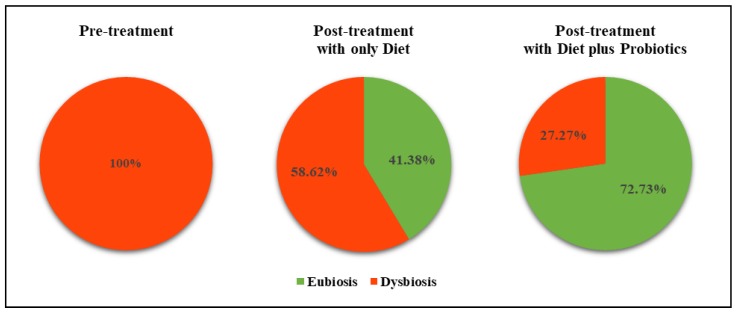
Pre- and post-treatment percentage distribution of eubiosis or dysbiosis condition, stratified for type of treatment (low-Ni diet alone or combined with probiotics).

Overall, at three months of treatment with a low-Ni diet alone, the percent of patients who shifted to eubiosis was 41.38%, while when the low-Ni diet was associated with the consumption of appropriate probiotic formulation, the profile strongly ameliorated with 72.73% of patients in eubiosis (Figure 6). The univariate logistic regression model showed that the targeted probiotic therapy, together with the dietary approach, increased the probability of reaching the eubiosis (OR= 3.77, IC 95% 1.14–12.47; *p* = 0.029). With regard to the gender parameter, despite males (*n* = 9, 17.65%) being much less represented than women (*n* = 42, 82.35%), some observations should be reported. Six male patients were included in the fermentative dysbiosis group while three were in the mixed dysbiosis group. Results obtained from the three males with fermentative dysbiosis after the low-Ni diet alone showed eubiosis in two of the patients, while no significant post-treatment changes of urinary indican were seen in the one patient with grade III dysbiosis. Of the three patients who followed the adjuvant treatment with probiotics, one patient reached eubiosis, while a grade I or III fermentation dysbiosis persisted in two patients. It should be noted that the male with grade III dysbiosis was overweight (BMI = 25) and, after treatment with probiotics, had a significant drop in the urinary levels of indican (from 137 to 54 mg/L), thus indicating an improvement in the intestinal condition. On the other hand, treatment with diet alone in 14 women with fermentative dysbiosis determined the appearance of eubiosis in five patients, five patients showed a shift towards a lower grade dysbiosis, and four patients showed the persistence of the pre-treatment dysbiosis level. Adjuvant treatment with probiotics resulted in a marked improvement in all 13 females, 10 of which reached the eubiosis condition and three patients showed a lower grade dysbiosis. Of note, all the three male patients with mixed dysbiosis (grade II and III) were obese grade-2 (BMI ≥ 30). Two patients followed the low-Ni diet alone and, even though post-treatment levels of indican and skatole decreased up to 50%, the initial degree of dysbiosis persisted. Of interest, the only male patient with mixed dysbiosis treated with diet plus probiotics switched from grade-III dysbiosis to the eubiosis condition. On the other hand, out of 13 women with mixed dysbiosis, 11 patients, including two overweight (BMI = 25), responded to treatment by switching to a lower grade dysbiosis or eubiosis. In particular, with the low-Ni diet alone, a shift to a lower grade dysbiosis or eubiosis could be observed, respectively, in four out of eight and three out of eight women, while the same degree of dysbiosis persisted in one out of eight female patients, although with a reduction in urinary marker levels. Among the five females with mixed dysbiosis treated with diet plus probiotics, four reached the condition of eubiosis while one persisted in the state of dysbiosis after treatment.

Comparing age classes built on the median age (*n* = 25 <37 years old; *n* = 26 ≥37 years old), significant lower values of pre-treatment indican dosages were found in the youngest age group (25 vs. 34 mg/L; *p* = 0.029) while no significant differences for post-treatment values were shown using the Wilcoxon rank-sum test (10 mg/L for the youngest vs. 11 mg/L for the oldest: *p* = 0.709). Lower values of pre- and post-treatment urinary skatole levels were also found in the youngest age group (16 vs. 23 g/L, *p* = 0.559; 6 vs. 10 g/L, *p* = 0.109, respectively), even though the differences were not statistically significant. It was therefore evident that the <37-year-old subjects had a less pronounced level of pre-treatment dysbiosis than those ≥37 years old. Of interest, both groups on average reached the condition of eubiosis after treatment with a low-Ni diet alone or combined with probiotics. About age-specific changes, taking into account either dysbiosis or treatment types, in both age groups the consumption of probiotics was found to be more effective than diet alone. In particular, in the younger group with fermentative dysbiosis (14), the shift towards a lower grade of dysbiosis or eubiosis was observed in 83.33% of subjects treated with probiotics (5/6) compared to 62.5% of the low-Ni diet alone group. In older subjects, while diet alone improved the intestinal condition in 77.78% of patients (7/9), after probiotic treatment 90% of subjects shifted to eubiosis or lower grade dysbiosis (9/10). Similarly, in younger subjects with mixed dysbiosis (9), adjuvant treatment with probiotics resulted in a reduction in the dysbiosis grade or eubiosis in 100% of patients (4/4) compared to 60% with diet alone (3/5). In the oldest patients with mixed dysbiosis (7), treatment with probiotics resulted in the achievement of the eubiosis condition in 100% (2/2) while the low-Ni diet alone led to an improvement in the dysbiotic condition in 80% of the subjects (4/5).

Of interest, the benefits of the low-Ni diet alone or combined with adjuvant therapy with probiotics were maintained until about 4–6 weeks after the end of treatment. In fact, after this time the pre-treatment clinical symptoms gradually reappeared in all patients, returning to the initial levels, thus indicating that the intrinsic individual risk factors did not allow the stability of positive effects.

## 4. Discussion

Considering that dietary Ni can alter the equilibrium of the symbiotic intestinal microbiota, the use of probiotic supplementation to restore the correct balance of the microbial community can be a successful adjuvant strategy to treat patients affected by SNAS. In the present study, we have analyzed the effectiveness of the combination of a low-Ni diet with the supplementation of a specific probiotic, selected on the basis of the dysbiosis type. First of all, confirming what some groups have previously reported [5,8,10,11,13,41], we want to emphasize that the low-Ni diet was very effective in reducing the clinical symptoms associated with SNAS, resulting in a significant increase in skin and oral Ni-tolerance levels. The assessment of urinary marker levels of gut microbiota functional imbalance, i.e., indican and skatole, allowed us to identify the type of dysbiosis in the 51 patients with SNAS, thus helping to accurately prescribe the most appropriate probiotic formulation. The first consideration is the prevalence of fermentative dysbiosis cases followed by mixed dysbiosis ones within the SNAS patients enrolled in the study. Based on the values of urinary markers, just two out of 51 patients were found to suffer from putrefactive dysbiosis. The high homogeneity of age, sex, and oral and skin Ni-sensitivity of our SNAS patient group allows us to conclude that, in this specific condition, the small bowel microbiota could be the predominantly perturbed one. In general, gut dysbiosis has been prevalently associated with altered concentrations of Lactobacilli and/or Bifidobacteria [25,27,28,29,31,33,35]. In particular, higher microbial-derived indican levels, indicative of fermentative dysbiosis and also associated with mixed dysbiosis, have been attributed to the reduction of several strains of Lactobacilli [23]. Our results showed that indican urinary values were above the normal range in 96% of SNAS patients, thus suggesting that Lactobacilli administration could be effective in reducing levels of SNAS-associated dysbiosis or even shifting towards eubiosis. In patients where the skatole level was also above the normal values, thus indicating a cover-up of putrefactive dysbiosis along with the fermentative (mixed dysbiosis), treatment was chosen with a probiotic formulation containing both Lactobacilli and Bifidobacteria strains. Of note, while a low-Ni diet alone significantly reduced Ni sensitivity and the level of dysbiosis, on the other hand, the probiotic adjuvant therapy led to a higher percentage of patients who shifted to a lower dysbiosis grade or eubiosis when compared to diet alone. Our results also indicated that the initial dysbiosis grade was higher in the population with above-average age (≥37 years) than in younger subjects (<37 years old). Of note, treatment with low-Ni diet alone or in association with probiotics led to an eubiosis condition in both age groups. With regard to the gender parameter, even if the characteristics of our sample of patients with 42 women and only nine men did not allow us to draw conclusions supported by a statistical point of view, in general we found that low-Ni diet treatment alone or combined with probiotics led to an improvement in dysbiosis conditions in 66.7% of male patients and 90.47% in females. This different trend could be attributed to the high frequency of obese or overweight subjects among males (4/9 patients, 44.4%) compared to that observed in the female sample (4/42 patients, 9.52%). Overweight or obese people in our patient sample accounted for just 13.7% (four males and three females). Recent studies reported an increased presence of Ni allergy, pooling together allergic contact dermatitis and SNAS, in obese patients and a possible link between obesity, hormonal dysregulation, and Ni allergy/exposure [42,43]. The authors conclude that toxic and immune-mediated effects of Ni may synergistically play a role in the genesis of obesity and hormonal impairment. Taking into account that microbial changes in the human gut are considered a factor involved in obesity development in humans, the hypothesis that Nickel-induced dysregulation of the host microbiota, intestinal barrier integrity and immune response may be the origin of the metabolic and inflammatory profile associated with obesity appears intriguing and is also supported by the benefits in obese individuals derived by consumption of selected prebiotics and/or probiotics [44]. A study on the effect of the low-Ni diet in addition to adjuvant treatment with probiotics could clarify the causal relationship between the harmful effects of Ni and the onset of obesity. In this regard, a recent study reported a prevalence of nickel allergy in IBS and the beneficial effects of a low-Ni diet on gastrointestinal symptom control, quality of life, and psychological status of patients [41]. In addition, also for the IBS condition, probiotics might be able to exert beneficial influences on several pathogenetic pathways of disease, including the restoration of altered gut microbiota by increasing the number of beneficial bacteria (Bifidobacteria and Lactobacilli among others). This would reduce the number of pathogens because of competition, and consequently the decrease of inflammation associated with the proliferation of pathogenic bacteria, changes in the metabolism of biliary salts, and the restoration of a normal colonic fermentation [45].

Altogether, our results strongly suggest that targeted probiotic therapy, together with an appropriate dietary approach, is an effective tool in correcting the intestinal dysbiosis associated with the SNAS condition and the consequent breakdown of the gut epithelial barrier. The specific mechanisms of action underlying the benefits of a probiotic treatment other than the normalization of perturbed intestinal microbiota communities also include direct effects on intestinal epithelial mucosa and modulation of the immune system [46,47,48]. Of interest, through in vitro models the direct beneficial effects of selected probiotics on damaged intestinal epithelial barrier have been recently reported by our group [49,50]. In this context, it may be useful to investigate the effect of appropriate probiotic administration on intestinal barrier function other than the dysbiosis level in SNAS patients.

While several studies have reported the beneficial effects of oral consumption of probiotics on some allergic diseases, including atopic dermatitis, allergic airways diseases, urticaria, and milk protein allergy [34,39,48,51], to date just one study is available in the literature on the effects of probiotic supplementation in SNAS patients [13]. In this work, Randazzo et al. have reported preliminary findings suggesting that the treatment of patients with SNAS with *L. reuteri* (10^8^ CFU per day) during a low-Ni diet led to an increase of intestinal lactic acid bacteria diversity and reduced gastrointestinal symptoms related to meals. Although with different approaches, our study, conducted on a higher number of cases with different treatment protocol either in terms of treatment period or daily dosages of probiotic products, and characterizing the type and degree of dysbiosis on each patient, supports the conclusions of Randazzo et al. and provides additional elements of knowledge, mainly in relation to the choice of probiotic to be administered on the basis of the individual dysbiosis type. Additional studies should be developed to identify the mechanisms underlying intestinal dysbiosis associated with SNAS as well as to define treatment with probiotics according to protocols that take into account the diversity of patients and, at the same time, predict common methodological approaches useful to define the status and functionality of the gut microbiota of SNAS patients or with other diseases. In fact, in addition to the ability of probiotics to restore a healthy gut microbiota and intestinal barrier integrity, it is also important to emphasize the contribution they can make to the immune system in a hypersensitivity condition such as SNAS. In general terms, the beneficial effect of probiotics on allergies has been often associated with enhancement of Treg cells as well as suppression of Th2 and Th17 responses [39,47,52]. In addition, new experimental approaches and technologies provided information on the influence of metabolites generated from dietary nutrients and selected probiotic strains on immune tolerance mechanisms [34]. The allergy-protective action of probiotics mainly consists of regulation of innate immune system functions, Foxp3^+^ Tregs, immunoglobulin A, and intestinal epithelial permeability [34,53,54,55,56,57,58,59]. Based on these findings, the improvement of the clinical symptoms and systemic manifestations in our patients after a low-Ni diet and probiotic treatment could be associated with the recovery of good functionality of the gut microbiota/gut system, allowing the restoration of physiological mechanisms of immune tolerance altered in the SNAS condition. In this regard, we are currently conducting a study aimed at clarifying whether the beneficial effect of probiotics on SNAS can lead, directly or indirectly, to the restoration of balance in immune cell populations predominantly involved in the pathogenesis of SNAS.

Overall, our results also underline the need to overcome the still widespread idea of considering the administration of probiotics as a generic integrative therapy. In this way, the appropriate probiotic for the treatment of a specific gut microbial picture can be suggested with the objective of formulating an increasingly personalized therapy. Finally, since the properties of the employed strain determine the efficacy of the treatment in each pathology, maintenance of the activity, viability, and growth efficacy of the probiotic upon technologic treatment should be verified. Indeed, the emerging evidence that manufacturing procedures may affect the efficacy and safety of probiotics imposes a special focus on choosing the probiotic to be administered [38,39,47,49,60,61,62,63,64].

Thus, pending stricter regulation of the quality and safety controls of probiotic products, a careful selection of the probiotic agent, standardization of the dose, and detailed characterization of the beneficial effects are crucial when considering the use of a probiotic for the dietary management of serious diseases.

In conclusion, dysbiosis is a compositional and functional alteration in the microbiota in individuals with disease compared with healthy subjects, and can feature a loss of beneficial microorganisms, i.e., Lactobacilli and Bifidobacteria, an expansion of potentially harmful microbes, and/or a loss of overall microbial diversity. Emerging evidence supports the hypothesis of dysbiosis as the first factor in the development of alterations in the intestinal barrier and immune system responsible for the occurrence of several allergic diseases, including SNAS. Besides exhibiting wide host diversity in composition, the gut microbiota is greatly affected by diet as well as host genetics, age, and sex [44]. The host body gets several benefits from a healthy microbiota strengthening of gut integrity, food metabolism, protection against virulent pathogens, and regulation of innate immunity. Many disorders have been linked to an altered microbiota with low diversity and lack of butyrate-producing bacteria in comparison to healthy individuals, both conditions frequently associated with immune and metabolically related diseases. Whether this disturbance in the microbial community is the cause or effect of a loss in the homeostasis remains to be determined. Nevertheless, there is good evidence that an altered microbiota can contribute to the pathophysiology of food allergies [34]. Being that gut microbiota composition and features in the SNAS condition are still largely unexplored, further investigations are needed to understand and exploit the microbial ecosystem of patients suffering from SNAS. Moreover, metabolomic studies will also provide important insights in the pathogenesis of SNAS, allowing researchers to establish microbiota causative involvement and creating the basis for implementing personalized medicine protocols. The multi-omic study of the relationship between the microbiome and SNAS may lead to defining the necessary associations and causality relationships for the subsequent transfer to clinical practice.

## 5. Limitations of the Study

Our study had several limitations. Firstly, being an observational study, the results are conditioned by various biases and structural limitations. Thus, our results should to be confirmed with randomized controlled clinical trials. In the era of personalized and precision medicine, an ideal approach would have been to define a personalized therapy based on specific individual alterations in the host microbiota, both in terms of relative abundance and metabolic activity. In our preliminary study, we relied on the urinary markers as general indicators of dysbiosis type to individuate the commercially available probiotic formulations potentially able to restore the Lactobacilli community in cases of fermentative dysbiosis and Lactobacilli plus Bifidobacteria in those patients with mixed dysbiosis. Even though our findings provide additional elements of knowledge about the association of SNAS and gut dysbiosis, patients’ intestinal microbiota in terms of structure and function as well as gut permeability pre- and post-treatment should be analyzed to verify the treatment success.

## Figures and Tables

**Table 1:** Reference intervals for the dysbiosis grades.

**Skatole**
0–10 μg/L (normal value)
11–20 μg/L (grade I dysbiosis)
21–40 μg/L (grade II dysbiosis)
>40 μg/L (grade III dysbiosis)
**Indican**
0–10 mg/L (normal value)
11–20 mg/L (grade I dysbiosis)
21–40 mg/L (grade II dysbiosis)
>40 mg/L (grade III dysbiosis)

**Table 2:** Characteristics of the patient samples stratified by dysbiosis type.

	Total *n* = 51	Fermentative *n* (%) 33 (64.71)	Putrefactive *n* (%) 2 (3.92)	Mixed *n* (%) 16 (31.37)	*p*-Value
Age, median (IQR)	37 (28–49)	38 (28–51)	32.5 (29–36)	34.5 (28.5–46)	0.689 *
Sex, *n* (%)					0.799 **
Males	9 (17.65)	6 (18.18)	0 (0.00)	3 (18.75)	
Females	42 (82.35)	27 (81.82)	2 (100)	13 (81.25)	
Ni-OST (mg), median (IQR)	1.8 (1.5–4)	1.75 (1.5–4)	1.5 (1.25–1.75)	2 (1.5–5)	0.405 *
Patch test, median (IQR)	2 (1–2)	2 (1–2)	2.5 (2–3)	2 (1–2.5)	0.472 *
Skatole (µg/L), median (IQR)	5 (2–13)	2 (1–5)	14 (11–17)	22.5 (12.5–37)	<0.001 *
Indican (mg/L), median (IQR)	28 (18–42)	31 (23–42)	6 (3–9)	30 (16.5–42.5)	<0.048 *

* Kruskal–Wallis test; ** χ^2^ test.

**Table 3:** Absolute and percent distribution of pre-treatment levels of skatole and indican

**Skatole**	***n* (%)**
0–10 μg/L (normal value)	33 (64.71)
11–20 μg/L (grade I dysbiosis)	9 (17.65)
21–40 μg/L (grade II dysbiosis)	6 (11.76)
>40 μg/L (grade III dysbiosis)	3 (5.88)
**Indican**	***n* (%)**
0–10 mg/L (normal value)	2 (3.92)
11–20 mg/L (grade I dysbiosis)	13 (25.49)
21–40 mg/L (grade II dysbiosis)	22 (43.14)
>40 mg/L (grade III dysbiosis)	14 (27.45)

**Table 4:** Comparison of pre- and post-treatment levels of urinary dysbiosis markers and Ni-OST.

	Pre-Treatment	Post-Treatment	*p*-Value *
Skatole (µg/L), median (IQR)			
Putrefactive	14 (11–17)	4 (1–7)	0.157
Mixed	22.5 (12.5–37)	9.5 (5–11)	<0.001
Indican (mg/L), median (IQR)			
Fermentative	31 (23–42)	10 (9–20)	<0.001
Mixed	30 (16.5–42.5)	11.5 (8–16.5)	0.001
Ni-OST (mg), median (IQR)	1.8 (1.5–4)	4 (3.5–5)	0.002

* Wilcoxon signed-rank test.

**Table 5:** Absolute and percent distribution of patients pre- and post-treatment with only diet or diet plus probiotics and comparisons of results obtained between patients who shifted to eubiosis or lower grade dysbiosis.

Groups	Diet		*p*-Value Pre-Post	Diet + Probiotics		*p*-Value Pre-Post	*p*-Value Post-Post
	Pre	Post		Pre	Post		
Shift to eubiosis, *n*. (%)	0 (0.00)	12 (41.38)	<0.001 *	0 (0.00)	16 (72.73)	0.031 *	0.026 **
Shift to a lower dysbiosis grade, *n*. (%)	29 (100.00)	17 (58.62)		22 (100.00)	6 (27.27)		
Fermentative, *n*. (%)	17 (58.62)	12 (41.38)	0.062 *	16 (72.72)	6 (27.27)	0.002 *	0.380 **
Mixed, *n*. (%)	10 (34.48)	5 (17.24)	0.063 *	6 (27.27)	0 (0.00)	0.031 *	
Ni-OST (mg), median (IQR)	1.80	4.00	0.031 ***	1.75	4.00	0.028 ***	0.765
	(1.38–5.00)	(3.00–5.00)		(1.70–3.00)	(3.50–5.00)		

* McNemar test; ** Fisher’s exact test or χ^2^ test; *** Wilcoxon signed-rank test; Wilcoxon rank-sum test.
